# Anticancer Drug Camptothecin Test in 3D Hydrogel Networks with HeLa cells

**DOI:** 10.1038/srep37626

**Published:** 2017-02-01

**Authors:** Jun Liang, Xiuzhi Susan Sun, Zhilong Yang, Shuai Cao

**Affiliations:** 1 College of Packaging and Printing Engineering, Tianjin University of Science and Technology, Tianjin, China; 2 Department of Grain Science and Industry, Kansas State University, Manhattan, Kansas, United States of America; 3 Department of Biological and Agricultural Engineering, Kansas State University, Manhattan, Kansas, United States of America; 4 Division of Biology, Kansas State University, Manhattan, Kansas, United States of America

## Abstract

Development of a biomimetic 3D culture system for drug screening is necessary to fully understand the *in vivo* environment. Previously, a self-assembling peptide hydrogel has been reported; the hydrogel exhibited physiological properties superior to a 3D cell culture matrix. In this work, further research using H9e hydrogel with HeLa cells was carried out considering H9e hydrogel’s interaction with camptothecin, a hydrophobic drug. According to AFM images, a PGworks solution triggered H9e hydrogel fiber aggregation and forms a 3D matrix suitable for cell culture. Dynamic rheological studies showed that camptothecin was encapsulated within the hydrogel network concurrently with peptide self-assembly without permanently destroying the hydrogel’s architecture and remodeling ability. Fluorescence measurement indicated negligible interaction between the fluorophore part of camptothecin and the hydrogel, especially at concentration 0.25 and 0.5 wt%. Using a dialysis method, we found that H9e hydrogel could not significantly inhibit the diffusion of camptothecin encapsulated inside the hydrogel matrix. In the cell culture experiment, HeLa cells were simultaneously embedded in the H9e hydrogel with the initialization of hydrogelation. Most importantly, cell viability data after camptothecin treatment showed responses that were drug-dose dependent but unaffected by the H9e hydrogel concentration, indicating that the hydrogel did not inhibit the drug.

The use of the cell-based model is still the main method for *in vitro* drug efficacy screening to more expediently determine optimal drug dosage in cancer therapy. The traditional two-dimensional (2D) cell based model has been limited by its low prediction efficiency compared to animal models, because of lack of multiple physical (e.g. stiffness, porosity and stress/strain), chemical cues (e.g. morphogens) and micoenvironmental elements (e.g. supporting extracellular matrix) resulting in various physiological performance issues^1^,^2^,^3^,^4^,^5^,^6^,^7^. Hence scientists and industries are switching to three-dimensional (3D) cell based models that are more accurately mimicking the native extracellular matrix (ECM) for *in vitro* cell culture^8^,^9^. Several matrix systems have been available for 3D cell cultures including PEG-fibrin hydrogel^10^, hyaluronic acid^11^, low-viscocity bioink^12^, fibrinogen and polyethylene Glycol^13^, etc., and among those, peptide hydrogel system becomes attractive due to its high water contents, good biocompatibility and using convenience.

The novel PepGel (H9e) peptide have been invented and serials of work have been done to explore its potential usage^14^,^15^. The peptide can form a solid physical hydrogel through *in vitro* assembly under conditions that mimic physiological conditions (i.e., neutral pH, 37 °C). Physical gelation under physiological conditions allows 3D homogeneous cell encapsulation. Moreover, h9e hydrogel displays shear-thinning and repeatedly reversible sol-gel transfer properties that enable pipette transfer of cell cultures^14^.

Drug diffusion in the 3D hydrogel network is an important factor in drug efficacy screening tests^16^. Some peptide hydrogels possess very strong adhesion to small molecules, making them potential carriers for controlled release^17^,^18^; however, for drug efficacy tests in 3D cell cultures, strong affinity between drugs and matrices can have negative effects. The matrix must have suitable diffusion properties in order to give the tested drug sufficient access to cells, and strong binding of drug molecules onto the hydrogel matrix could inhibit drug diffusion and reduce the drugs’ effects, creating inaccurate experimental results^17^. Thus, the interaction between H9e peptide and drugs must be studied, to ensure h9e hydrogel does not inhibit the effect of drug^19^.

In this research, we examined the suitability of the peptide hydrogel as a 3D system to evaluate the efficacy of small hydrophobic anticancer drug molecules. We selected camptothecin as the drug molecule because it exhibited remarkable anticancer activity in preliminary clinical trials but low solubility in aqueous solution^19^,^20^,^21^,^22^. We focused on the interaction of h9e hydrogel nanofibers and the selected drug along with drug diffusion in the 3D system. An luminescence method was used to detect the interaction between camptothecin and h9e hydrogel, atomic force microscopy (AFM) was used to image the hydrogel formation, and a rheometer was used to study the drug’s influence on hydrogel strength and sol-gel recovery properties. UV-absorbance was performed to detect the diffusion of camptothecin as a function of hydrogel concentration. HeLa cells were used as a model to test the performance of camptothecin in various concentrations of the h9e hydrogel. A combination of physical and biological analysis proved that h9e hydrogel was a potential promising 3D cell culture for hydrophobic drug camptothecin.

## Results and Discussion

### Camptothecin interaction with h9e hydrogel

**Figure 1:**
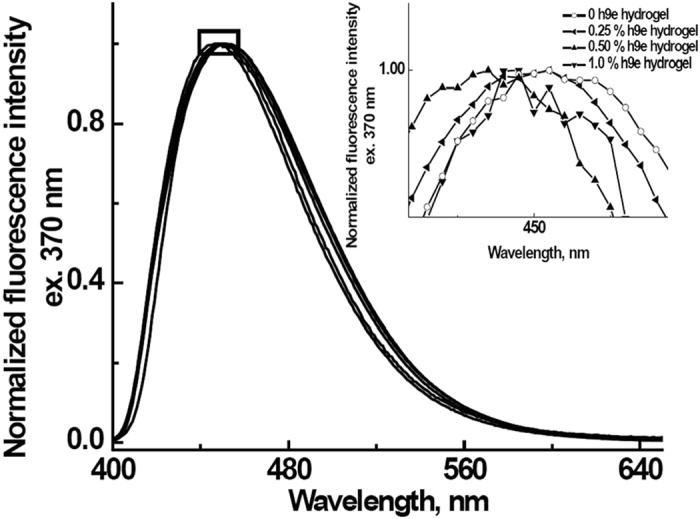
Fluorescence spectra from camptothecin encapsulated in h9e hydrogel. Insert shows the enlarged square area.

Camptothecin is a naturally fluorescent molecule. The fluorescence spectrum, a measure of the average energy of emission, is related to the polarity and flexibility of the polyphenolic side-chain environment^23^. Permana and his coworkers reported that the interaction of camptothecin with carbon nanotubes generated an over- 20 nm shift at most in the fluorescence peak from camptothecin^24^. In this study, we used fluorescence spectroscopy to study microenvironment changes of camptothecin in H9e hydrogel. Figure 1 overlays normalized fluorescence bands of 2.5 mM camptothecin in 0.25, 0.5, and 1% of h9e hydrogel and the control sample of 25 μM camptothecin in 100 mM sodium bicarbonate solution. As shown in the insert of Fig. 1, an introduce of 1% h9e hydrogel only leads to a 3-nm shift in the peak of fluorescence from camptothecin, indicating that the interaction between camptothecin and h9e peptide fibers is minor.

### Atomic force microscopy(AFM)

**Figure 2:**
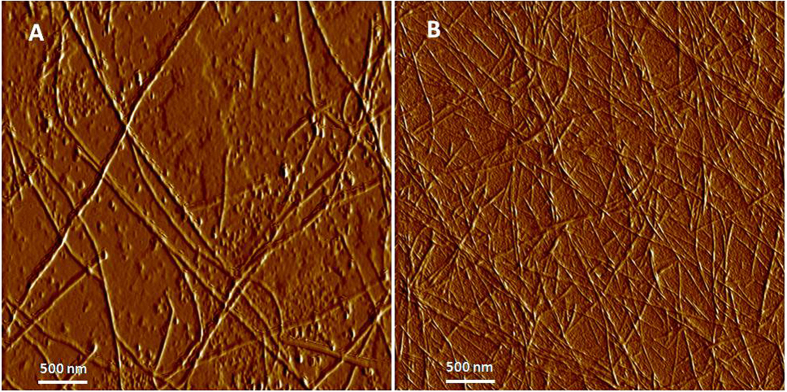
Tapping mode AFM height images of H9e hydrogelin 100 mM sodium bicarbonate solution (**A**) and 100 mM sodium bicarbonate solution containing PGworks (**B**).

Peptide hydrogel can be formed by adjusting pH^25^, temperature^26^, or chemical cross-linkers^27^, depending on the peptide backbone structure. Specific protein-peptide interactions have been used to direct self-assembly of peptide nanowires into micrometer-sized crystalline cubes^28^. For gelation in this study, h9e hydrogel was triggered by PGworks solution with bovine serum albumin (BSA) as the main functional component. AFM showed that the peptide can self-assemble into nanofibers with a lot of fragments, even without the PGwork trigger solution (Fig. 2A); however, these nanofibers did not form stable 3D networks as hydrogel, most likely due to the lack of strong interactions between peptides at this concentration. After the addition of PGworks solution, the fragments disappeared and longer fibers were observed, forming extensive cross-linking points, leading to the formation of 3D networks for hydrogel (Fig. 2B).

BSA, a widely studied protein, has been used as a model protein for diverse biophysical, biochemical, and physicochemical studies^29^,^30^,^31^. The unique 3D structure of BSA, determined by X-ray crystallography, is composed of three domains (I, II, and III) that confer a heart-shaped molecular structure; the structure forms a large hydrophobic cavity that acts as a hydrophobic binding site^32^,^33^. BSA’s ability to bind to a variety of hydrophobic ligands, such as fatty acids^34^, bilirubin^35^, warfarin^36^, tryptophan^37^, and several dyes, has been reported. For this study, the turning function of GSII in the peptide backbone of h9e played a key role in hydrogel formation by interacting with hydrophobic force^15^, and the added BSA enhanced the hydrophobic interaction by acting as a linker that binds multiple sequences of GSII through hydrophobic force.

### Oscillatory rheology

**Figure 3:**
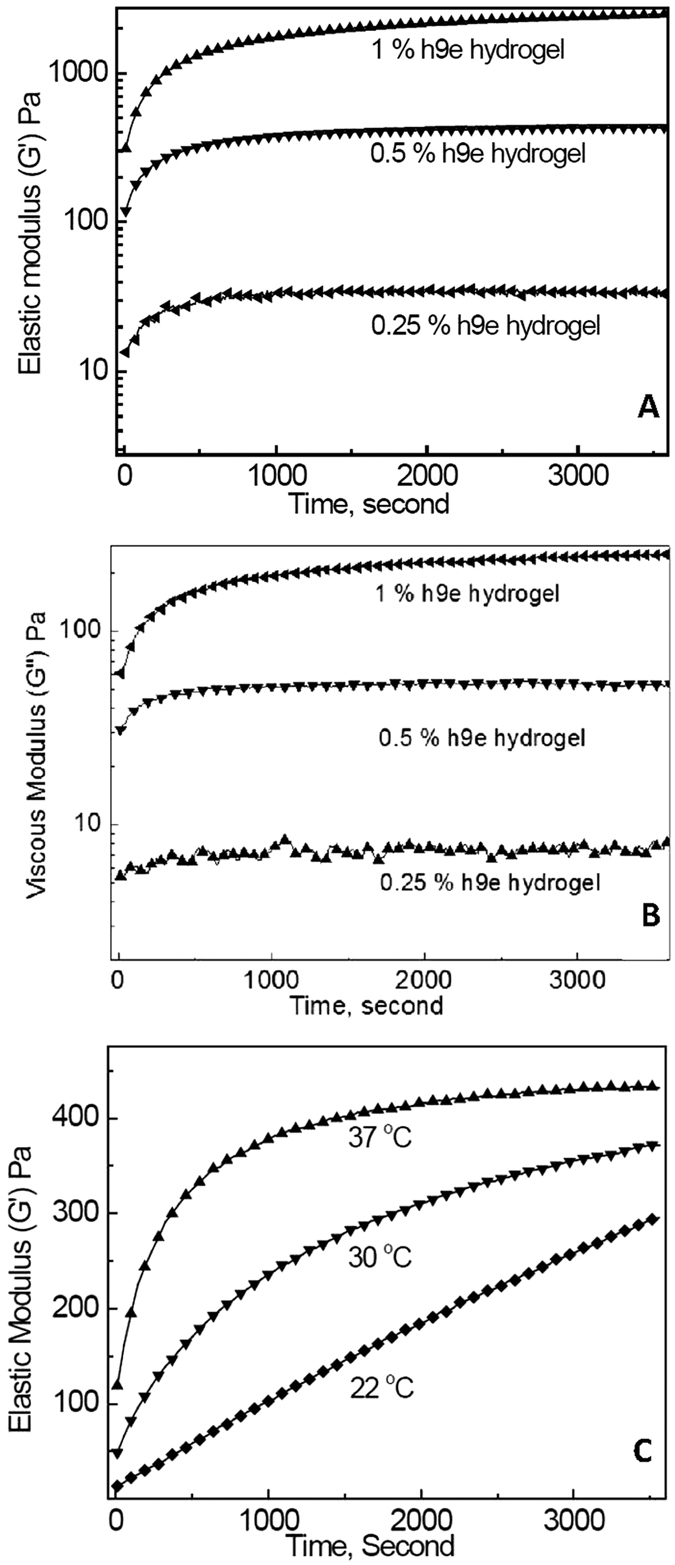
Storage (G′) and loss (G′′) moduli of 0.25, 0.5, and 1 wt% H9e hydrogel in DMEM containing 10% FBS during hydrogelation at 37 °C (**A,B**) respectively)), and G′ of 0.5 wt% at 22, 30, and 37 °C (**C**).

Oscillatory rheology measurements were performed to observe gelation kinetics of 0.25, 0.5, and 1.0 wt% h9e hydrogels at 37 °C (Fig. 3A and B). In order to study the effects of temperature on gel formation, we treated 0.5 wt% h9e hydrogels at various temperatures (37, 30, and 22 °C) (Fig. 3C). Measurements were carried out immediately after h9e hydrogel was mixed with the trigger solution. G′ for 0.25, 0.5, and 1.0 wt% H9e hydrogel at 37 °C were 35, 432, and 2480 Pa, while for G′′, the values are 7, 53, and 220, respectively, 60 minutes after initial gel formation. For the 0.5% h9e hydrogel at various temperatures, G′ increased more quickly at a higher temperature than at a lower temperature. In another group, results of a sample encapsulated with camptothecin indicated that this small hydrophobic drug did not obviously change the strength of h9e hydrogel (data not shown).

**Figure 4:**
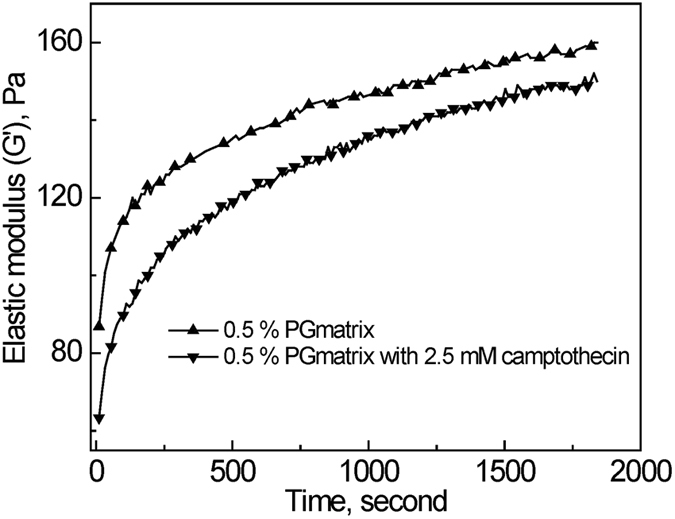
Storage modulus (G′) recovery at 37 °C after shear–thinning treatment for 0.5 wt% H9e hydrogel prepared in DMEM containing 5% H9e hydrogel without (**A**) and with (**B**) 2.5 mM camptothecin.

The influence of camptothecin on strength and gelation recovery after shear-thinning treatment (Fig. 4) was also evaluated. Freshly prepared 0.5% h9e hydrogel with and without 2.5 mM camptothecin was kept in the plate at 37 °C for 60 minutes. Steady shear (100 s^−1^ for 10 s) was subsequently applied to the samples to mimic shear forces applied to the samples in a syringe injection process. For the sample without camptothecin, observed moduli were 86 Pa immediately after the shear-thinning treatment and 160 Pa 60 minutes after shear-thinning. For the sample loaded with 2.5 mM camptothecin, observed moduli were 63 Pa immediately after shear thinning and 150 Pa 60 minutes after shear-thinning. Essentially, no significant effect of camptothecin was found on gelation, shear-thinning, or the gel recovery process. In another work, we have done the shear-thinning treatment with trigger calcium and observed the moduli recovery over 2 hours, and similarly, only minor effect of camptothecin on the shear-thinning properties of H9e hydrogel was found (data not shown).

Results reported in this study are in agreement with results reported by Huang *et al*.^15^ H9e hydrogel with and without camptothecin immediately displayed solid-like properties after shear thinning and recovered within a short time period.

### Camptothecin diffusion

**Figure 5:**
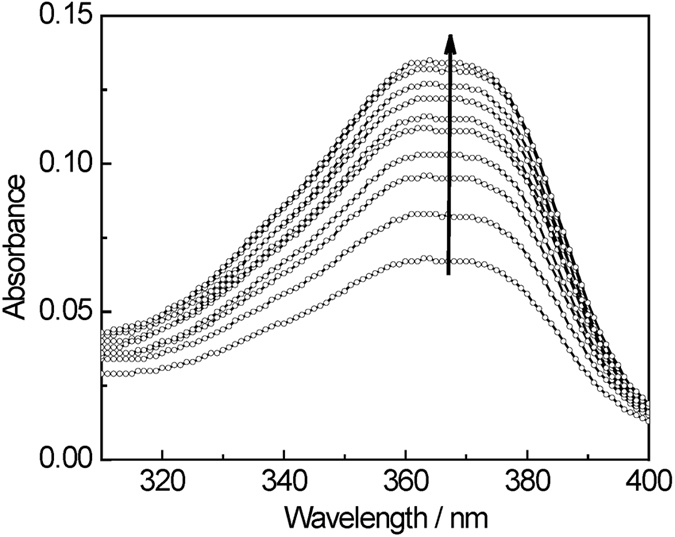
Absorbance spectra of the surrounding solution in the release experiment of camptothecin from H9e hydrogel. Measurements were taken at 0.5-h intervals initially and then later increased to 13 h. Lowest and highest curves were obtained at 0.5 and 21 h, respectively.

**Figure 6:**
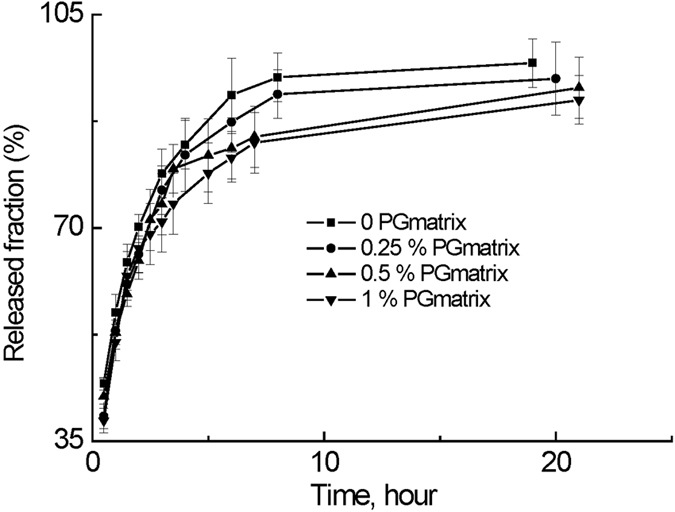
Fraction release of camptothecin as a function of time from various concentrations of H9e hydrogel at 37 °C.

The most common methods currently used to study drug diffusion in gel matrices are sample separation and dialysis^38^. In this study, we introduced the drug-loaded h9e hydrogel into a dialysis tube in a flask containing Phosphate-buffered saline (PBS) buffer as the outer solution. We monitored the drug release for a specified time by measuring UV absorbance of the camptothecin diffused into the flask; representative data for 0.5 wt% H9e hydrogel in 37 °C are plotted in Fig. 5. Diffusion studies with the dialyzer showed that approximately 80% of the camptothecin diffused from the dialyzer into the outer solution within 5 hours at 37 °C (Fig. 6); the rates of camptothecin release from peptide hydrogel concentrations of 0.25, 0.5, and 1% were comparable, suggesting that the passage of camptothecin through the dialyzer° occurs independently of H9e hydrogel concentration and that peptide binding is not a concern in camptothecin diffusion.

### HeLa cell viability

**Figure 7:**
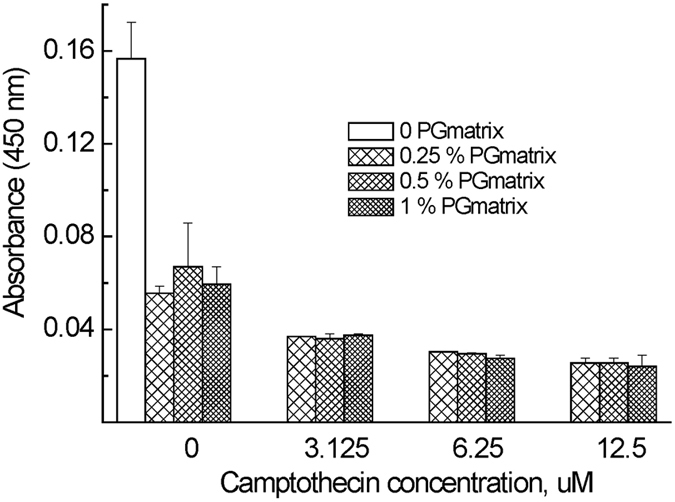
Effect of camptothecin on the absorbance of 450 nm obtained from CCK-8 assay. Error bars are +/− standard error (n = 3). The *p*-value of each group samples against another two groups with different camptothecin concentration was under 0.0001, while in the same group, *p*-value of each sample against another two samples with different h9e hydrogel concentration was above 0.50.

**Figure 8:**
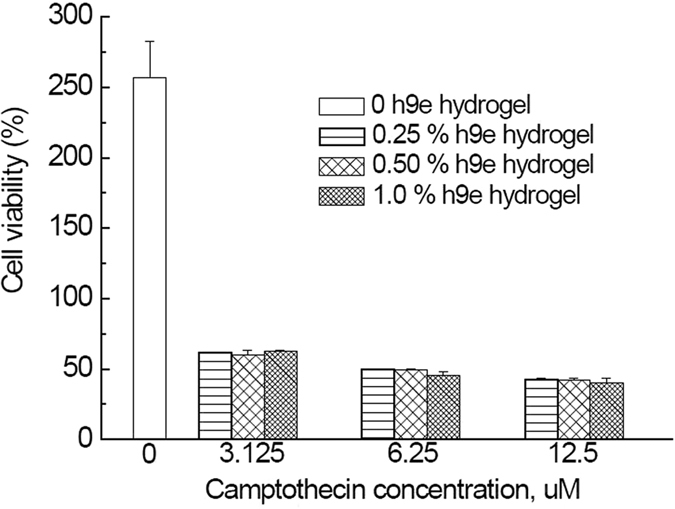
Viability of HeLa cells treated with 3.125 uM, 6.25 uM, and 12.5 uM camptothecin in 0.25, 0.5, and 1 wt% hydrogels for 3 days. Error bars are +/− standard error (n = 3). The *p*-value of each group samples against another two groups with different camptothecin concentration was under 0.0001, while in the same group, *p*-value of each sample against another two samples with different h9e hydrogel concentration was above 0.50.

The ability of camptothecin to induce apoptosis in HeLa cells by microRNA-125b-mediated mitochondrial pathways has been previously reported^39^. After treating Hela cells in h9e hydrogel for 3 days with various concentrations of camptothecin, we found a dose-dependent decrease in cell proliferation indicated by the absorbance of 450 nm obtained from CCK-8 assay (Fig. 7) and cell viability (Fig. 8). The final DMSO in the 2D cell culture and 3D h9e hydrogel was 0.34 (w/v)%; previously it has been reported that when the concentration of DMSO in the cell culture was below 0.5%, no obvious cytotoxicity was observed in Hela cells^40^, and because we have added an equal concentration of DMSO in all samples, the impact of DMSO on the differences of result can be ignored. Various concentrations of h9e hydrogel treated with the same amount of camptothecin showed comparable cell viability, implying that h9e hydrogel didnot inhibit the effects of camptothecin for killing HeLa cells. This result was in accordance with our fluorescence measurement and diffusion results, which demonstrated that h9e hydrogel did not inhibit the diffusion of camptothecin. In addition, 2D and 3D cultures promoted Hela cell proliferation over time; however, the proliferation rate indicated was higher for 2D culture than for 3D culture samples (Fig. 7). Similar phenomena were also found in other 3D culture systems^41^ and other tumor cells in H9e hydrogel^14^. Because the increase of h9e hydrogel concentration didnot lead to decreased cell viability, we suggest that the proliferation difference for samples in 2D and 3D cultures was caused by growth pattern change.

## Conclusions

H9e hydrogel showed a negligible binding effect with the hydrophobic drug camptothecin used in this study as a model for small hydrophobic drugs. Therefore, gel strength and recovery properties of h9e hydrogel were not significantly influenced by the addition of camptothecin, and the camptothecin molecule diffused well in h9e hydrogel. *In vitro* experiments with HeLa cells have shown that camptothecin induced dose-dependent cell viability, and the increase in hydrogel concentration did not significantly impact the drug efficacy test, suggesting that h9e hydrogel did not influence biological activities of cells induced by camptothecin. The combined physical and biological results clearly indicate that h9e hydrogel is an appropriate 3D cell culture matrix to test the effect of camptothcin. Our research team is exploring the performance of h9e hydrogel with protein drugs, hydrophilic drugs with large molecular weight, BSA-linked drugs, and other hydrophobic drugs.

## Materials and Methods

### Hydrogel preparation

H9e hydrogel kit (PepGel PGmatrix) at a concentration of 2 wt% H9e hydrogel peptide solution and PGworks solution were provided by Dr. Sun’s lab in the Department of Grain Science, Kansas State University. For hydrogelation, H9e hydrogel solution, was added into 100 mM sodium bicarbonate solution, or Dulbecco Minimum Essential Medium (DMEM, Sigma Chemical, St. Louis, MO) containing 5% PGworks protein based solution, or DMEM containing 10% Fetal Borne Serum (FBS); both are used to trigger the hydrogel formation. The hydrogel formed within 30 minutes at room temperature with a final peptide concentration of 0.25, 0.5, or 1 wt%, respectively.

### Fluorescence

The fluorescence technique was used to evaluate the interactions between camptothecin and hydrogel peptide nanofibers, using a PAM-101–103 fluorometer (H. Walz, Germany). Emission spectra ranged from 400 to 650 nm with an excitation wavelength of 370 nm. To minimize background scattering, the h9e hydrogel peptide was dissolved in 100 mM sodium bicarbonate. Camptothecin was dissolved into dimethyl sulphoxide (DMSO) to prepare 50 mM of stock solution, and aliquots of the drug stock solution were added to the hydrogel-forming solution to obtain a final concentration of 2.5 mM for camptothecin and 5% (v/v) for DMSO. Final camptothecin concentrations for this experiment were 2.5 mM in 0.25, 0.5, and 1 wt % h9e hydrogel, respectively. The mixture solutions were kept in the fluorescence cells for 30 minites before measurements were carried out. For the control, 2.5 μM camptothecin in 100 mM sodium bicarbonate solution containing 5% (v/v) dimethyl sulfoxide (DMSO) without h9e hydrogel was used to determine spectroscopic characteristics of camptothecin in aqueous solution. Spectra were normalized at the emission peak.

### Atomic force microscopy

Tapping mode AFM images were collected using an Innova Atomic Force Microscope (Bruker, Camarillo, CA) under ambient conditions. The 2 wt% h9e hydrogel solution was diluted with DI water to a final concentration of 0.004 wt%. Samples were prepared by spotting 20 μl solution with and without PGworks solution on 12-mm mica discs that were then settled at room temperature for at least 3 hours until the solution dried completely. Drive frequency of the silicon tip was tuned and fixed at 200–250 kHz before engagement. Amplitude profiles were obtained using Nanoscope Analysis software V1.40. All collected images were flattened before further analysis.

### Rheological tests

Gelation of the h9e hydrogel as function of time was determined by using the C-VOR 150 rheometer system (Malvern Instruments, Malvern, Worcestershire, United Kingdom) with a 20-mm-diameter parallel plate geometry and 500-mm gap size. Storage moduli (G′) was used as the gel strength. Samples with and without camptothecin were prepared as previously described with DMEM containing 5% PGworks solution. Immediately after the PGworks solution was added, 200 μl of gel-forming solution was placed on the measuring system. A single frequency (1 Hz) and a steady shear strain (1%) were used for the test. For shear-thinning experiments, samples were subjected to 100 s^−1^ shear for 10 seconds, after which the strain was reduced to 1%. Gel recovery of G′ as a function of time (1 Hz frequency, 1% strain) was subsequently monitored for 1 hour.

### Camptothecin diffusion test

Drug diffusion was observed by measuring the drug released from the gel to the outer solution. Aliquots of the drug in DMSO were added to the hydrogel-forming solution to obtain a final concentration of 2.5 mM for camptothecin and 5% (v/v) for DMSO. Samples containing 0-mM camptothecin were prepared using DMSO without camptothecin. A Float-a-Lyzer (1000 KD MWCO, 1-mL capacity, regenerated cellulose membrane) was purchased from Spectrum Labs (Rancho Dominguez, CA). We used the 1000 KD MWCO membrane because the MWCO’s aperture is large enough to allow the h9e hydrogel peptide and camptothecin molecules to pass through. Then 1 mL of 0.25, 0.5, and 1 wt% hydrogel containing 2.5 mM camptothecin in 100 mM sodium bicarbonate was introduced into the inner tube of the dialyzer, which was then placed into a 500-mL glass cylinder containing 300 ml phosphate-buffered saline (PBS) as release media (outer solution). The outer solution was continually stirred at 130 rpm using a small magnetic stir bar to prevent formation of an unstirred water layer at the membrane/outer solution interface. Sampling the contents of the outer solution at periodic intervals assessed drug diffusion to the outer solution at 37 °C. At certain time intervals, 1 ml of solution was taken from each release system (i.e., the outer solution) for UV absorbance measurements, and the same volume of a PBS buffer, previously kept at 37 °C temperature as the individual release system, was added in order to maintain a constant volume of outer solution.

UV absorbance measurements were carried out on a UV-1650PC spectrophotometer (Shimadzu, Kyoto, Japan). The absorbance peak at 370 nm was recorded for all measured solutions. A release fraction was calculated by the function (I_t_/I_∞_), where I_t_ is the absorbance of drug released at time t, and I_∞_ is the absorbance of drug released at time infinite; the latter marks the measured absorbance of total camptothecin in the 1 ml h9e hydrogel dissolved in 300 ml PBS. Released fractions were averaged from three replicates for each sample, and mean values and standard errors were determined each time.

### Cell culture and drug efficacy tests

HeLa cell suspension in DMEM media was prepared following commonly used methods. Cells were detached from 2D cell culture flask by using a trypsin treatment and cell pellet was collected by centrifugation at 1000 rpm for 5 minutes, and re-suspended in DMEM. Cell concentration was counted using an Auto Cellometer Mini (Nexcelom Bioscience, Lawrence, MA). Aliquots of HeLa cells solution were then added into H9e hydrogel DMEM containing 10% FBS with a final peptide concentration of 0, 0.25, 0.5, and 1 wt%. Then 100 μl of cell mixture solution (3 × 10^4^ cells /wall) was seeded into a 96-wall culture plate (Becton Dickinson Labware, Franklin Lakes, NJ) and placed in an incubator (Nuair, Playmouth, MN) in a humidified 5% CO_2_ atmosphere at 37 °C for approximately 30 minutes. After gelation, 10 μl of DMEM with 5% (v/v) DMSO containing 0, 50 uM, 100 uM, and 200 uM camptothecin was added on top of the hydrogel in order to obtain camptothecin concentrations of 0 μM, 3.125 μM, 6.25 μM, and 12.5 μM, respectively. Then 50 μl of DMEM was carefully added to the top of the hydrogel to prevent the gel from drying. All measurements were made at least in triplicates, and the plates were incubated for 3 days.

A CCK-8 assay was used to determine cell viability after each treatment, and 10 μL of CCK solution was added to each wall. After 4 hours of incubation, absorbance at 450 nm was collected on a microplate reader (mQuant, Bio-Tek) and corrected by subtracting the background signal from a wall containing only 160 μl DMEM with 10% FBS. Absorption intensities were averaged from three replicates for each sample and normalized by cells seeded in H9e hydrogel cell culture without being treated with camptothecin (control) in order to obtain cell viability. Mean values and standard errors were determined each time. For group comparisons, the one-way layout ANOVA with duplication was applied. Significant differences in the mean values were evaluated by the student’s unpaired *t*-test. A *p* value of less than 0.05 was considered significant.

## Additional Information

**How to cite this article**: Liang, J. *et al*. Anticancer Drug Camptothecin Test in 3D Hydrogel Networks with HeLa cells. *Sci. Rep.* **7**, 37626; doi: 10.1038/srep37626 (2017).

**Publisher's note:** Springer Nature remains neutral with regard to jurisdictional claims in published maps and institutional affiliations.
